# Perfect single-photon sources

**DOI:** 10.1038/s41598-023-47585-9

**Published:** 2024-02-01

**Authors:** Sana Khalid, Fabrice P. Laussy

**Affiliations:** Faculty of Science and Engineering, University of Wolverhampton, Wulfruna St, Wolverhampton, WV1 1LY UK

**Keywords:** Quantum optics, Single photons and quantum effects

## Abstract

We introduce the *gapped coherent state* in the form of a single-photon source (SPS) that consists of uncorrelated photons as a background, except that we demand that no two photons can be closer in time than a time gap $t_\text{G}$. While no obvious quantum mechanism is yet identified to produce exactly such a photon stream, a numerical simulation is easily achieved by first generating an uncorrelated (Poissonian) signal and then for each photon in the list, either adding such a time gap or removing all photons that are closer in time from each other than $t_\text{G}$. We study the statistical properties of such a hypothetical signal, which exhibits counter-intuitive features. This provides a neat and natural connection between continuous-wave (stationary) and pulsed single-photon sources, with also a bearing on what it means for such sources to be perfect in terms of single-photon emission.

## Introduction

The single-photon source (SPS) is a cornerstone in photonics and for quantum-optical technology^1–4^. It describes light that is produced photon by photon, thereby fighting their urge (as bosons) to pile-up together, as they do when left in thermal equilibrium^5^. Since Glauber’s theory of photon correlations^6^, the quantity of choice for the SPS is the second-order coherence function $g^{(2)}(\tau )$ that quantifies the density of two-photons separated in time by $\tau$. A SPS should in particular be such that no two photons are ever found together, so $g^{(2)}(0)=0$ is wanted. In practice, though, $g^{(2)}(0)$ is “only” much smaller than unity, with $g^{(2)}=1$ describing uncorrelated photons, whose proximity is left to chance. The failure to be exactly zero allows for cases where $g^{(2)}(0)\ll 1$ while $g^{(3)}(0)\gg 1$, the latter quantity describing three-photon coincidences (this could also be true for any $g^{(n)}$). In contrast, if $g^{(n)}(0)=0$ (exactly) then all $g^{(m)}(0)$ with $m\ge n$ must also be zero. While it certainly may seem strange that a source which suppresses strongly its two-photon coincidences does not compulsorily also suppress higher-order ones, this is nevertheless a possibility (as long as $g^{(2)}(0)$ is not *exactly* zero). This illustrates how subtle photon correlations can be Refs.^7,8^ and how difficult it is to define a good criterion for SPS^9^. In this text, we consider hypothetical signals that will allow us to precise such notions.

## The concept of perfect single-photon emission

It is clear from our introduction that $g^{(2)}(0)=0$ *exactly*, is a requisite for the perfect single-photon source. What would such an ideal source look like, if we could further conceptualize its attributes? There, the dynamics of emission and detection enters the picture. Indeed, one could imagine a steady-state SPS where, at each and every moment of time, the field has a single photon. This would constitute a continuous (all-times) yet still quantized (one-photon) field. This is a strange, ultimate quantum field. The emission of an incoherently-pumped two-level system quenched in its excited state approaches such a scenario in the limit of infinite pumping rate: the system is constantly kept excited as it constantly emits single-photons. This is a highly-pathological object which, for instance, is impossible to simulate as a point process with “clicks” by, say, quantum Monte Carlo procedure^10^, since the time steps should be vanishing. This makes obvious the fundamental problem that one should undertake an exact measurement in time to observe only a single photon from such a source. The slightest holding-up of time will accumulate several of the single photons and spoil the SPS character. A manifestation of such a pathological feature is that the luminescence spectrum of such a source is flat (photons are emitted at all the frequencies) with a vanishing height, as the area is unity, corresponding to the population of the emitter (which is maintained in its excited state). Nevertheless, this continuous flow of single photons is indeed such that $g^{(2)}(0)=0$, exactly. In this case, the emissions at all﻿—i.e., including infinitely large—frequencies, correspond to the vanishing times of the single-photon emissions.

**Figure 1:**
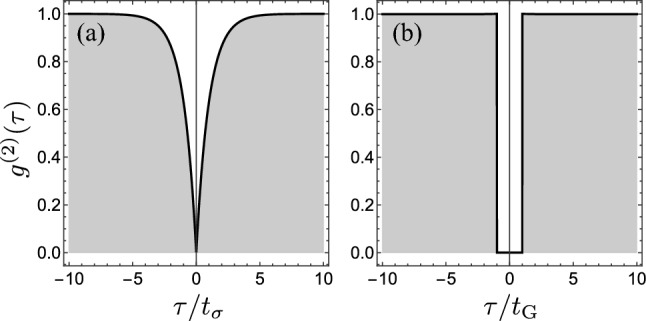
Glauber’s second-order correlation function $g^{(2)}(\tau )$ for (**a**) the incoherently-driven two-level system, in units of $t_\sigma =1/\gamma _\sigma$ and (**b**) a gapped single-photon source, with vanishing $\gamma t_\text{G}$.

This shows that, while it is straightforward to have a dynamical system that results in $g^{(2)}(0)=0$ exactly, this comes as an unphysical limit for the optimum emission rate: the optimum perfect single-photon source is pathological (here note that we use two similar adjectives to refer to different attributes: brightness and multi-photon suppression). Turning to finite pumping rate $P_\sigma$, the emission of a two-level system, with decay rate $\gamma_\sigma$, still features exactly $g^{(2)}(0)=0$ and the spectrum becomes physical: a Lorentzian of width $\gamma _\sigma +P_\sigma$ and area $P_\sigma /(\gamma _\sigma +P_\sigma )$. We have insisted on the extreme (optimum), unphysical case above as it actually captures the key problem in realizing a single-photon source: the perfect time-resolution for the detection is still needed also for the physical SPS. Although an incoherently-pumped two-level system spaces its successive emitted photons in time, and despite the suppression indeed of their proximity as compared to a Poisson process, detection in time will always accidentally collect various photons together, unless there is no time uncertainty whatsoever, i.e., the detection is temporally point-like. This brings forward the problem of the photon correlations in time, through $g^{(2)}(\tau )$ where $\tau$ is the time-delay between any two detected photons. For a two-level system, $g^{(2)}(\tau )=1-\exp (-\gamma_\sigma |\tau |)$ as shown in Fig. 1a. The theory of photo-detection^11^ shows that any measurement comes with a finite time-resolution^12^ and since $g^{(2)}(\tau )=0$ exactly, only for $\tau =0$ exactly, any overlap with nonzero times bring us back to an imperfect $g^{(2)}(0)$. In frequency space, this means that although emitting with a physical Lorentzian spectrum, one will not get perfect antibunching unless one detects all the frequencies, which is in practice impossible, since Lorentzian have fat tails and so, regardless of the bandwidth of the detector, some photons will always escape and, ironically, this results in bunching^11^. Such fundamental aspects tend to be underappreciated and relegated to technical limitations from the setup, instruments and/or the samples. While those certainly still take the largest share of the problem, even if they could be mitigated or even suppressed altogether, one would still face the fundamental problem we have just discussed. Now that we have highlighted the situation with paradigmatic two-level SPS (this is further discussed in Refs.^9,12^), we can cut the Gordian knot and turn to a scheme that would, in principle and at a fundamental level, provide a perfect SPS.

**Figure 2:**
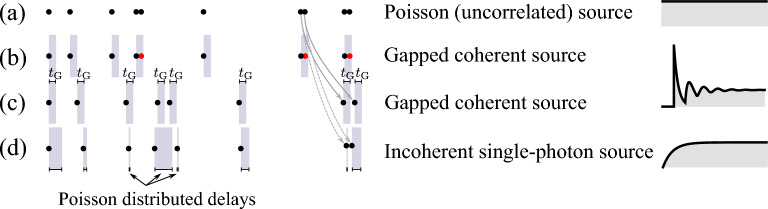
A point process view of photon emission: in (**a**), a typical sampling of ten points randomly distributed in time, with no correlations, describing photons from a coherent state. In (**b,c**), two possible constructions are shown of a gapped coherent source from the backbone Poisson stream, formed by (**b**) removing photons closer to their predecessor than the time gap $t_\text{G}$, here stripping off the red points, or by (**c**) adding the time gap as an extra “spacer” between every photon from the original stream. If such a repulsion is not rigid but also randomly distributed according to a Poisson distribution, as shown in (**d**), instead of a gapped coherent source, one recovers the emission from a conventional incoherently pumped two-level system. Although infrequent, Poisson repulsion can result in some photons bunching, due to the Poisson bursts, as shown for the 7th and 8th photons in stream (**a**), that may remain very close to each other as shown in (**d**), in contrast to their gapped counterpart (**c**).

## Gapped coherent state

In this text, we do not consider quantum-dynamical mechanisms to produce light but directly streams of photons as events in time (time series) and study their general mathematical structures following various desiderata that one might require from a SPS (from now onward, it will therefore be convenient to understand SPS as “single-photon stream”)^13^. In particular, this allows us to consider what, following our previous discussion, constitutes clearly a “perfect SPS” even in the face of imperfect detectors: one needs to open a gap in time between successive photons, to enforce, by construction, no-coincidences not only at $\tau =0$ but at all times within the gap, i.e.,

1 $$\begin{aligned} g^{(2)}(|\tau| <t_\text{G})=0. \end{aligned}$$

Outside of the gap, we may expect photons to have no correlations and thus

2 $$\begin{aligned} g^{(2)}(|\tau| \ge t_\text{G})=1, \end{aligned}$$

as shown in Fig. 1b. This can be obtained from a Poisson background as the simplest case—which also corresponds to the popular coherent state—by opening a time gap in its emission, and thus we refer to this hypothetical signal as the “*gapped coherent state*”. For such a stream of photons, no two photons are ever found in the same time interval $t_\text{G}$, regardless of where this is sampled. One possible way to construct it as a pointlike process is, starting from a random (Poisson) sequence (Fig. 2a), to remove every instance that, if leaving it there, would result in more than one photon in the time interval $t_\text{G}$ (Fig. 2b). Another equivalent way, thanks to the properties of Poisson processes, is to add a time gap $t_\text{G}$ after each photon (Fig. 2c). Poisson emission being independent at all times of what happened previously, the addition “by-hand” of a gap does not alter the probability of emission after the gap. In all cases, the sources should be normalized to their emission rate which, if the original, underlying Poisson process has emission rate $\gamma$, is then, by opening a gap of duration $t_\text{G}$, given by:

3 $$\begin{aligned} \gamma _\text{G}\equiv{\gamma \over 1+\gamma t_\text{G}}. \end{aligned}$$

There are various ways to obtain this result. From the numerically more efficient scheme in Fig. 2c), one sees that for a total number of events (“clicks”) $N_c$, emitted at a rate $\gamma$ so that the last photon is detected at time $t_\text{last}=N_c/\gamma$ (first at $t_\text{first}=0$), adding a time gap subsequent to each photon leaves us with an emission rate $\gamma _\text{G}=N_c/(t_\text{last}+N_ct_\text{G})$ which is, dividing numerator and denominator by $t_\text{last}$, Eq. (3). Obviously, opening a gap lowers the intensity, a point to which we shall return later on.

Such a gap, for which $g^{(2)}(\tau )=0$ not only at $\tau =0$ but for some finite time window, clearly solves the issue of finite-time resolution: as long as our detector is more accurate in time than $t_\text{G}$, it will never accidentally collect two or more photons at once. A gap is precisely what makes a superconductor a perfect conductor^12^, although measurements also come with imperfections for an electric current as well. One can still cancel the resistivity exactly (meaning that deviations from zero cannot be measured). Photons being precisely quanta, so discrete quantities, it should be expected that one could similarly achieve $g^{(2)}(0)=0$ exactly for light, meaning that one could generate a perfect stream of single-photons without ever observing more than one at a time for as long as we count. So far, instead, one could “only” considerably suppress, but not eliminate completely, coincidences^14,15^ (this can still be claimed mathematically^16^).

**Figure 3:**
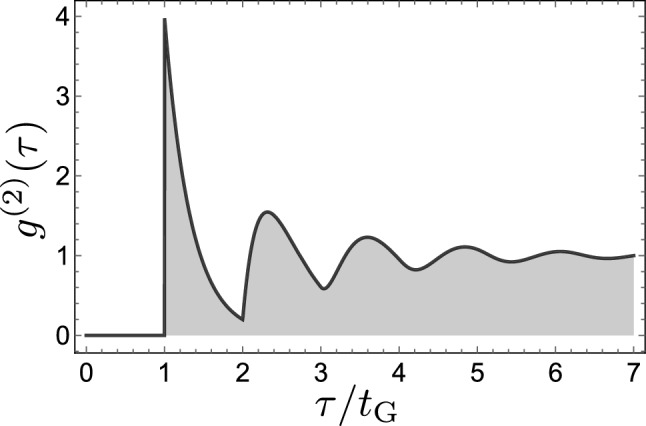
Glauber’s second-order correlation function $g^{(2)}(\tau )$ for a gapped single-photon source with $\gamma t_\text{G}=3$ (shown for positive $\tau$ only, the function is symmetric). The single-photon gap is followed by strong correlations of the bunching type which are damped in time.

An interesting property of such a gapped stream of photons is that, while it satisfies Eq. (1) by construction, later photons do not “remain” uncorrelated with the one that opens the gap, i.e., we do not have Eq. (2) as one could expect but, instead:

4 $$\begin{aligned} g^{(2)}(\tau ) = (1+\gamma t_\text{G})e^{-\gamma (|\tau |-t_\text{G})}\sum ^{\infty }_{n = 0} \frac{\big [\gamma \big (|\tau | - (n+1)t_\text{G}\big )e^{\gamma t_\text{G}}\big ] ^{n}}{n!}{\mathbbm{1}}_{[(n+1)t_\text{G}, \infty [} (|\tau |), \end{aligned}$$

where ${\mathbbm{1}}_A(t)$ is the indicator function, i.e., it is 1 if $t\in A$ and 0 otherwise. An example of such correlations is shown in Fig. 3. There is a superbunching peak immediately after the gap and then damped oscillations with a frequency approximately given by Eq. (3). This is an interesting feature given that $g^{(2)}(\tau )$ measures a density of two-photon events, regardless of whether photons are successive ones or if there are others in between them: it correlates each photon to every other photon. Therefore, that a photon at $\tau =t_\text{G}$ “becomes” correlated with a photon at $\tau =0$ by the mere fact that those in between have been removed could be surprising. Quantum opticians know well that $g^{(2)}(\tau )$ is insensible to losses and whether all photons are detected or only a tiny amount of them does not change their measurement (only the time they need to acquire it with the noise-to-signal ratio they want). Another way to describe this apparent paradox is that from an initially uncorrelated stream of photons, one can produce positive correlations of a bunching type by *removing* photons, and adding nothing.

The reason for these strong positive correlations can be linked to the Monty Hall problem^17^ that is a famous paradox whereby providing apparent useless information to a decision-problem turns out to alter its chance of success, when someone has knowledge of how to provide uselessly this information. Namely, in the Monty Hall problem, a player is given a choice between three doors, one of which conceals a prize. After selecting a door, someone with knowledge of which door hides the prize opens one of the two unselected doors (without prize). Should the player stick to their initial choice or switch to the remaining door to win the prize? Counter-intuitively, the player, who had initially a probability 1/3 to find the prize, now has probability 2/3 if they switch the door. This is surprising at first since, whatever the initial choice, it is always possible to open a loosing door, so no information seems to have been revealed.

In our case, enforcing the single-photon attribute in a given time-window would similarly seem to be unrelated to photon correlations elsewhere. Why would a photon from an initially uncorrelated stream become more likely to be detected immediately at the time at which we have been revealed that no other photons are present? It would indeed be the case that no such dramatic correlations occur if the subtracted photons would have been so by chance, and not deterministically (just like the Monty hall paradox breaks if the door is opened randomly). Then $g^{(2)}(\tau )$ would remain identity everywhere. We find, instead, that enforcing Eq. (1) comes at the price of propagating correlations at all times according to Eq. (4).

We now proceed to explain the origin for this result which, like the Monty Hall problem, although maybe counter-intuitive at first, is actually easily and unequivocally understood by working out the corresponding probabilities (in the Monty Hall case it is interesting that it generated considerable opposition, in particular as its popularization came from a woman^18^, and that even Erdös himself was not accepting the result^19^). In our case, one can observe that the stronger correlations are in the second time-window following the gap one, i.e., for ${t_\text{G}\le t\le 2t_\text{G}}$, where one has, furthermore, a simple exponential decay. This is because in this window, we have one and only one photon (if there is no photon, then this does not contribute to signal as already pointed out, and there can be no two photons in a time window of width $t_\text{G}$ by construction). This photon remains Poisson distributed in its window since any possibly-removed photon was in the previous time window $t<t_\text{G}$ and the one left felt no influence from the memoryless nature of Poisson processes. We have therefore, in this window, the $g^{(2)}(\tau )$ of an heralded single photon (the “heralder” being the first detected photon which opens the gap window) which, if its own distribution of time emission is a simple exponential process, as is the case for random emission, also decays exponentially^20^. It is also important that no other photon be present in the second time window, as one would have otherwise an other scenario of an heralded *N*-photon emission which is multi-exponential^13^.

To work out the correlations at subsequent times, we follow the route opened by Reynaud^21^ of working out photon correlations from conditional probabilities of the emission processes, and by Kim et al.^22^ who further established the relationship between the waiting time distribution $w(\tau )$ between *successive* photons and Glauber’s $g^{(2)}(\tau )$ between any two photons:

5 $$\begin{aligned} \gamma _\text{G} g^{(2)}(\tau )=w(\tau )+(w * w)(\tau )+(w * w * w)(\tau )+\cdots , \end{aligned}$$

where * denotes convolution, i.e., for the second term of this infinite series:

6 $$\begin{aligned} (w * w)(\tau )\equiv\int _0^\infty w(\tau ')w(\tau -\tau ')\,d\tau ', \end{aligned}$$

where the upper limit for integration could have been stopped at $\tau$ since $w$ is zero for negative arguments, in which case this provides the probability of detecting a photon at $t=0$ and another one at $t=\tau$ with one and exactly one photon in between at time $t=\tau '$, averaged over all possible values of $\tau '$, i.e., this provides the waiting-time distribution $w_2$ for the second photon.

In our case, the waiting time distribution is a good starting point since this is simply for our gapped coherent source:

7 $$\begin{aligned} w(\tau )=\gamma e^{-\gamma (\tau -t_\text{G})}{\mathbbm {1}}_{[t_\text{G},\infty [}(\tau ). \end{aligned}$$

This follows again from the memoryless properties of Poisson point processes, and is an obvious consequence of the equivalent construction as sketched in Fig. 2c (the sum of two Poisson processes is also a Poisson process). Note that unlike $g^{(2)}(\tau )$ that measures the density of any two-photon events, the waiting-time distribution that quantifies successive events is actually intuitive.

The waiting-time distributions are thus obtained by computing Eq. (6) for Eq. (7), i.e.,

8 $$ w_2(\tau )= \gamma ^2\int _{0}^{\tau } e^{-\gamma (\tau '-t_\text{G})}e^{-\gamma (\tau -\tau '-t_\text{G})} {\mathbbm {1}}_{[t_\text{G},\infty [}(\tau '){\mathbbm {1}}_{[t_\text{G},\infty [}(\tau -\tau ')\,d\tau '. $$

This would be a simple convolution of exponentials and thus a trivial result, except that the indicator functions impose various restrictions on the time domains, namely, they require $\tau$ to be larger than $2t_\text{G}$ and $\tau '$ to remain between $t_\text{G}$ and $\tau -t_\text{G}$:

9 $$\begin{aligned} w_2(\tau \ge 2t_\text{G})=\gamma ^2\int _{t_\text{G}}^{\tau -t_\text{G}} e^{-\gamma (\tau '-t_\text{G})}e^{-\gamma (\tau -\tau '-t_\text{G})}\,d\tau ', \end{aligned}$$

so that finally, one has:

10 $$\begin{aligned} w_2(\tau )=\gamma ^2(\tau -2t_\text{G})e^{-\gamma (\tau -2t_\text{G})}{\mathbbm {1}}_{[2t_\text{G},\infty [}(\tau ). \end{aligned}$$

Subsequent cases are similarly obtained from the higher-order convolutions, corresponding to the successive single photons isolated in their respective gap windows. The general case is obtained as the *n*th convolution which can be calculated by recurrence:

11 $$ w_n(\tau )\equiv w^{*n}(\tau )=(w^{*(n-1)} * w)(\tau ), $$

with similar constrains on the domains of integration, now involving the product of indicator functions ${\mathbbm {1}}_{[nt_\text{G},\infty [}(\tau ){\mathbbm {1}}_{[t_\text{G},\infty [}(\tau -\tau ')$ to yield

12 $$\begin{aligned} w_n(\tau )=\gamma \int _{(n-1)t_\text{G}}^{\tau -t_\text{G}}w_{n-1}(\tau ')e^{-\gamma (\tau -\tau '-t_\text{G})}\,d\tau ', \end{aligned}$$

which provides the *n*th photon waiting time distribution

13 $$\begin{aligned} w_n(\tau )=\frac{\gamma ^n\big (\tau - nt_\text{G}\big )^{n-1}e^{-\gamma (\tau -n t_\text{G})}}{(n-1)!}{\mathbbm {1}}_{[nt_\text{G}, \infty [} (\tau ), \end{aligned}$$

from which our main result Eq. (4) is obtained through Eq. (5) (including for $t\le t_\text{G}$) as $g^{(2)}(\tau )=\gamma _\text{G}^{-1}\sum _{n=1}^\infty w_n(|\tau |)$. Note that the actual emission rate of the stream, so $\gamma _\text{G}$, provides the link between $g^{(2)}$ and $w$ in Eq. (5) while the original rate $\gamma$ from the underlying Poisson process enters in the waiting time distribution (7), so that the maximum amount of correlations, at the time gap is given by

14 $$\begin{aligned} g^{(2)}(t_\text{G})={\gamma \over \gamma _\text{G}}=1+\gamma t_\text{G}. \end{aligned}$$

This is also obtained directly from Eq. (4) as $\lim_{\tau\to t_\text{G}\atop\tau>t_\text{G}}g^{(2)}(\tau)$. Note also that $g^{(2)}(t_\text{G})$ is not actually defined, or presents a discontinuity there, since $\lim_{\tau\to t_\text{G}\atop\tau<t_\text{G}}g^{(2)}(\tau)=0$ and Eq. (14) really refers to the $\tau >t_\text{G}$ limit. The correlations are maximized by large values of $\gamma t_\text{G}$, which is also the mean number of photons in the time gap (before removing the extra ones in Fig. 2a). This brings us back to the Monty Hall problem where the amount of deterministically removed information is linked to the magnitude of the effect (an insightful argument from vos Savant was to generalize the game to a large number of doors^18^ making the probability to win by switching converge to one).

From these results, one can now work out the conditions to produce a SPS that satisfies Eqs. (1) and (2): this is obtained in the limit of no signal, since from Eq. (3) one must have $\gamma t_\text{G}=0$ or, for sizable $t_\text{G}$, a vanishing $\gamma$. A perfect SPS with no extra-correlations is thus one with very scarce signal: a dim laser (coherent or Poissonian) from which Poisson bursts are brushed off with the gap, that, in most cases, will do nothing as there are so few clicks anyway, but whenever two (or more) clicks would get close by chance, the extra ones are removed. In fact, Fig. 1b has been obtained in this way with $\gamma t_\text{G}=10^{-3}$ and is thus a physical $g^{(2)}$ (the departure to the ideal case is not visible to the eye).

## Pulsed single-photon source

Another limit to contrast to $\gamma t_\text{G}\rightarrow 0$ in Fig. 1b, is that where $\gamma t_\text{G}\gg 1$, in which case, although the signal is still, eventually, stationary, its correlations are so strong as to be reminiscent of the opposite regime of single-photon emission: that of pulsed excitation.

The pulsed SPS consists in generating each photon individually with pulses of excitation that drive the system in its excited state^23,24^. The ideal limit consists of a train of equally spaced photons with a Dirac comb of two-photon correlations:

15 $$\begin{aligned} g^{(2)}(\tau )=t_\text{G}\sum _{n\ne 0}\delta (\tau -nt_\text{G}), \end{aligned}$$

where the time gap is now externally imprinted by the laser pulses. The peak averages are normalized:

16 $$\begin{aligned} {1\over t_\text{G}}\int _\text{peak} g^{(2)}(\tau )\,d\tau =1. \end{aligned}$$

Considering fluctuations, or *jitter*, of each photon emission around their reference $nt_\text{G}$ emission time, according to a distribution *D*(*t*), we find:

17 $$\begin{aligned} g^{(2)}(\tau )=t_\text{G}\sum _{n\ne 0}(D \star D)(|\tau -nt_\text{G}|), \end{aligned}$$

where $\star$ represents this time the auto-correlation:

18 $$\begin{aligned} (D\star D)(\tau )\equiv \int _{0}^{+\infty } D(t)D(t+\tau )\,d t. \end{aligned}$$

**Figure 4:**
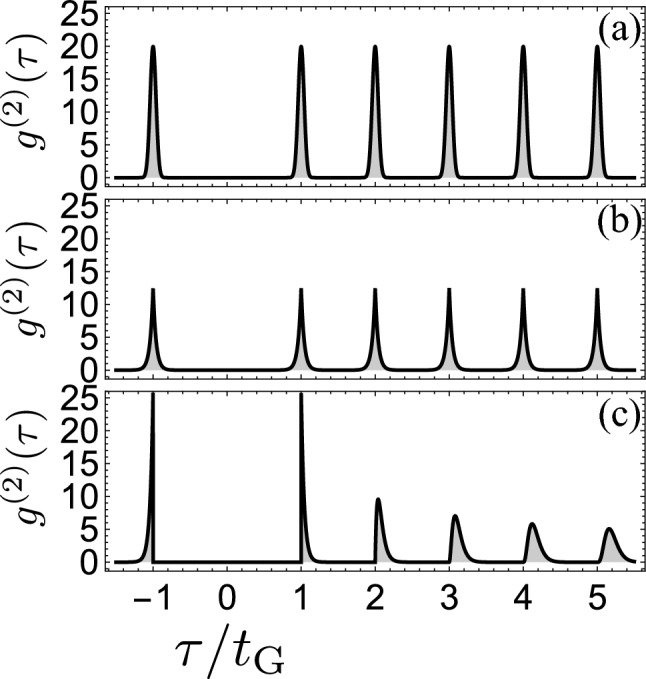
Two-photon correlations in (**a,b**) the pulsed regime and (**c**) the stationary regime for a gapped SPS with $\gamma t_\text{G}=25$. The pulses would eventually overlap, wash out and plateau to 1 in the latter case. In the pulsed case, the suppressed peak at $\tau =0$ is the only signature of two-photon suppression while the full structure is a testimony to that in (**c**).

We show, in Fig. 4 the cases of (a) normally-distributed fluctuations, i.e., with $D(t)={\sqrt{{2\gamma ^2}/{\pi }}{e^{-\frac{1}{2}(\gamma t)^2}}}$, and (b) of asymmetric exponential decay with $D(t)={\gamma e^{-\gamma t}}$ along with, in panel (c), Eq. (4) for $\gamma t_\text{G}\gg 1$. The resemblances are clear as well as the reason why: in such a case, the background Poisson signal is so strong that its gapping is, indeed, tantamount to creating a periodic array of single-photons since for a sufficiently bright signal, there is always a random photon close enough to the start of the next gapping window, after all those extra from the previous window have been trimmed-off. Clearly this produces a stream similar to a sequence of equally-spaced pulses. This is in fact another way to dissipate immediately the counter-intuitive features of the gapped stream: seeing it as a stationary version of the pulsed SPS, where the ordering is not externally imprinted, but internally by the gapping of the photons. It must have a finite coherence-time since, as for all stationary signals, $g^{(2)}(\tau )=1$ for $\tau$ large enough and so there must be a dissolving of the peaks into a flat background. The reason for this return to stationarity is due to the accumulated effect of time fluctuations for each peak in the gapped case while, when they come from external pulses, they are reset for each peak. This results in several differences, even in the strongly correlated region. Foremost, since the waiting time distribution Eq. (13) is normalized, in the limit where the peaks are greatly separated, they also fulfill Eq. (16). Figure 4c in fact shows the $w_n(\tau )$ in isolation. In stark contrast with the pulse case, however, there is a decay of the maxima of the peaks with an envelope for the comb. Such a decay can also be found in some pulsed-excitation schemes, but for a different reason. Santori et al.^25^ indeed observed (experimentally) a decaying envelope for the peaks, which they identified was caused by blinking, i.e., intermittency of the emission, a well-known problem of solid-state emitters. This furthermore concerns, precisely, the *areas* of the peaks, which decay, while in our case, the *maximum* of the peaks, that otherwise remain normalized, decay, until the peaks broaden to a point that they become unresolvable and a flat $g^{(2)}(\tau )=1$ is recovered (stationarity). Another striking difference is that pulsed autocorrelations feature symmetric peaks, since whatever shape the jitter distribution *D* takes, its auto-correlation Eq. (18) symmetrizes the photon coincidences, that can be equally delayed or postponed. This is seen in Figs. 4a,b that stem from both symmetric and asymmetric fluctuations. In contrast, the gapped SPS is markedly asymmetric, with photons being delayed only, since the gap is, by construction, impenetrable. As another consequence, the frequency increasingly lags in time.

## Discussion

Our starting point was the requirement of a stream of photons that ensures that never two photons be found together. Let alone other important attributes for many applications (such as indistinguishability or brightness^26–28^ that are crucial for applications^29^), this is a universal and basic requisite for a single-photon source. We have discussed how this becomes a fundamental problem for the paradigmatic cases of a two-level system spontaneously emitting, when including their detection—which is central in a quantum theory—as this two-photon suppression reduces to one-time only ($\tau =0$). Demanding such a suppression over a finite interval of time—a time gap—we found interesting properties of the resulting signal that shed light on the meaning and nature of single-photon emission. Foremost, we find temporal two-photon correlations, which we have described exactly. The phenomenology is interesting because such oscillations are commonplace with strongly-driven two-level systems, and are usually associated to Rabi dynamics. We find in contrast (not opposition), that regardless of the mechanism to produce photons, the mere presence of the gap condition Eq. (1) results in such oscillations as the emergence of an ordering of the photons in a sequence. This is a property to keep in mind when considering highly antibunched sources: such oscillations are the manifestation of single-photon emission that is so good that even in a stationary regime, it acquires some properties of the pulsed regime. This further shows that brightness, i.e., the intensity of the emission, that is not or not well captured by $g^{(2)}(\tau )$, is an important aspect of single-photon emission, and not only for considerations of signal but as a result of photon ordering. In contrast, indistinguishability appears to remain less connected to the problem of suppression of multiple photons, although in the case of an incoherent two-level system, we have shown that one can realize an ideal perfect SPS at the cost of a complete loss of indistinguishability. We also emphasized the counter-intuitive nature of $g^{(2)}(\tau )$ as opposed to other, but less popular, quantities such as the waiting-time distribution. Although the peculiarities we have focused on are quickly understood, it is easy to cook-up further counter-intuitive features. As another illustration of this, if in our gaping procedure one does not always remove the extra photons but does so with probability *p* only, instead of the perfect antibunching (1), one now produces actual coincidences for $|\tau |<t_\text{G}$ from the underlying uncorrelated stream, namely, $g^{(2)}(\tau )=(1-p)(p \gamma t_\text{G}+1)e^{- p \gamma |\tau |}$ which thus turns to bunching $g^{(2)}(0)>1$ for $p<1-\frac{1}{\gamma t_\text{G}}$. This is always possible (positive *p*) as long as $\gamma t_\text{G}\ge 1$, i.e., there should be more than one photon on average in the time gap. In this case, the maximum bunching is $(1 + \gamma t_\text{G})^2/(4\gamma t_\text{G})$ for the probability $p={1\over 2}(1-{1\over \gamma t_\text{G}})$ to remove photons closer than $t_\text{G}$. This tends to infinite bunching for 50% removal of the photons in the gap, in the limit of large $\gamma t_\text{G}$. Again, how removal of photons—this time probabilistic as opposed to systematic—from an uncorrelated stream, can result in arbitrarily large and genuine bunching (as opposed to antibunching), may be surprising at first. The reader should however be able to explain it and provide the full $g^{(2)}(\tau )$ for this case as well following our above discussion^13^.

Our approach also provides another and insightful picture of the standard SPS as realized by an incoherently-pumped two-level system: this can be understood similarly to the scheme of Fig. 2c but instead of “inserting” a constant time gap $t_\text{G}$ after each photon from the underlying uncorrelated stream, one instead inserts a Poisson-distributed delay (i.e., following the exponenial distribution with parameter $\gamma '$). Then one obtains the two-photon correlation function of the incoherent single-photon emitter $g^{(2)}_\text{2LS}(\tau )=1-\exp \big (-(\gamma +\gamma ')|\tau |\big )$ (we remind that $\gamma$ is the emission rate of the uncorrelated backbone emission) for a total emission rate of the SPS thus formed of $\gamma \gamma '/(\gamma +\gamma ')$ according to the arguments given to obtain Eq. (3). This provides a numerically easy and efficient way to generate such a stream^30^ obtained under either the condition $P_\sigma =\gamma$ and $\gamma _\sigma =\gamma '$ or the symmetric $P_\sigma =\gamma '$ and $\gamma _\sigma =\gamma$. Such a picture should also make even more clear why an incoherently-excited two-level system fails to provide perfect single-photons: its suppression of coincidences stems from the feeblest type of repulsion between photons, featuring indeed the highest probabilities for smaller delays and whose underlying emission is furthermore prone to Poisson bursts, as shown in Fig. 2a. This is the reason for the steep $g^{(2)}(\tau )$ and why its $\tau =0$ coincidences can never vanish entirely at a fundamental level: initially closely-spaced photons can be further unlucky to not be much separated by their Poisson repulsion. While this combination of events is indeed unlikely, it is doomed to happen over a sufficient large sampling (one instance is captured from actual simulation for the seventh and eighth photons in streams (a) and (d) of Fig. 2).

Our discussion also tampers down the statement that it “is not enough to have single-photon wavepackets to have single-photon light” but that one “need[s] in addition to know at which time each single-photon wavepacket is emitted”^31^. We have just exhibited a source from which one has no long-term knowledge of when each photon is to be emitted, but that, locally, is even more efficient than existing sources in suppressing coincidences.

The problem of how to actually engineer a quantum emitter that would produce such a gapped coherent state is another question. We want to highlight, however, that the joint subnatural linewidth antibunched sourced proposed in Ref.^32^ bears some resemblance to our idealized case and was indeed the motivation for this work. Namely, there is a plateaued $g^{(2)}(\tau )$ which, although not strictly zero, is still much flatter than the incoherently-driven two-level system. Furthermore, its emission is obtained at the price of a considerable drop in the signal, indeed removing—not extra photons in a time gap—but the coherent fraction of the signal, again bringing the question of brightness into the shape of the correlations. Contrasting this case—which is, this time, the result of an explicit dynamical model—with our previous discussion, highlights again how the imperfect single-photon character of the incoherently pumped two-level system stems from the feeble repulsion between photons. In contrast, a distribution of time delays that is minimum at $\tau =0$ (instead of maximum like for the incoherent SPS), e.g., of the Maxwell distribution type (from the kinetic theory of gases), does produce a plateaued $g^{(2)}(\tau )$ much like the one found in the coherently homodyned emitter^32^. This raises further questions on the nature and origin of antibunching in resonance fluorescence^33^ and indeed invites to design schemes to more strongly repel photons—qualitatively^34,35^ in the way each photon pushes away the next ones as described by $g^{(2)}(\tau )$—instead of “merely” quantitatively^36,37^—reducing $g^{(2)}(0)$—so as to eventually implement a perfect single-photon source.

Finally, our discussion of temporal correlations of photons relates to a popular problem in condensed-matter physics, namely, of the so-called pair correlation function *g*(*r*) for the distance *r* (in space), also known as the radial distribution function, of fluids^38^. This has long been known from the diffraction of X-rays to exhibit damped oscillations, with shapes that are reminiscent of those we have described above. Our result Eq. (4) is indeed found in this context in the extreme idealization for a liquid as noninteracting hard-spheres in one dimension (so “hard rods”). This has been derived by several authors using a variety of techniques, from probabilistic arguments of the Clausius or Smoluchowski type for the distribution of free paths of a molecule in a gas^39,40^, to integral equations derived from the partition function^38,41–43^, where Eqs. (1–2) are known as the “hole-correction approximation”. Our own solution is obtained from the probabilistic side, but invoking arguments of spontaneous emission from heralded photons instead of configurations of points distributed spatially on a finite line before taking the thermodynamic limit. Such direct eventual relationships bring the question whether thermodynamic concepts as well as results and techniques of statistical physics could shed light on photon correlations of increasingly complex types of quantum light, and if one should characterize photon streams as temporal Tonks gases, with an equation of state to relate their entropy with other macroscopic variables such as their pressure and compressibility, albeit, again, in time as opposed to in space. With this parallel, one could thus understand the case of Fig. 4c as a photon (temporal) crystal and that of Fig. 3 as its melting into a photon (temporal) fluid. Whether such parallels can benefit one or the other discipline remains to be ascertained but their conceptual proximity cannot be denied, and old results obtained under drastic approximations for fluids may well be resurrected as fundamental for photons which are intrinsically non-interacting and, temporally, one-dimensional.

## Summary and conclusions

We have taken a point-process approach to photon correlations, by focusing on the signal as “clicks” recorded in time. This has allowed us to formulate a concept of a perfect SPS in the form of a gapped coherent state, which is a Poisson (stationary) stream in which a gapping condition is enforced that no two photons are ever found closer than the time gap $t_\text{G}$. This ensure that $g^{(2)}(0)$ can actually be measured as exactly zero even with imperfect detectors or taking into account fundamental time uncertainties from the measurement, and thus also enforce the vanishing of coincidences of higher-numbers of photons (that are, counter-intuitively, possible to any degree if $g^{(2)}(0)$ is merely very small but nonzero). We have shown that this results in strong correlations in the form of oscillations that alternate bunching and antibunching, with, in particular, increased probabilities to find a photon immediately after the gap, followed by damped oscillations. We have explained the nature of the phenomenon and provided closed-form analytical expressions for it. We also identified various fundamental attributes in the nature of single-photon emission, including the constraints imposed by the signal (intensity) with the result of transposing our stationary source into one with attributes of pulsed emission, thereby establishing a fundamental connection between continuous-wave and pulsed single-photon sources. This allowed us to revisit the nature of the paradigmatic single-photon emission from the incoherent emission of a two-level system, that similarly introduces, not a rigid gap, but an exponentially distributed delay between photons. The relationship of the gapped coherent state to the recently proposed scheme of joint antibunched and subnatural-linewidth emission, and still other emitters able to realize other types of photon repulsions, as well as other phases of matter, could be fruitfully pursued.

## Data Availability

All data generated or analysed during this study are included in this published article.
